# Extrapelvic Endometriosis Involving Pudendal and Sciatic Nerve Causing Obturator Muscle Atrophy

**DOI:** 10.5334/jbsr.3657

**Published:** 2024-09-30

**Authors:** Ottavia Battaglia, Tjalina Hamerlynck, Pieter De Visschere

**Affiliations:** 1 Università degli Studi di Milano, Milan 20141, Italy; 2 Women’s Clinic Ghent University Hospital, Ghent, Belgium; 3 Department of Radiology and Nuclear Medicine, Genitourinary Radiology and Mammography, Ghent University Hospital, Ghent, Belgium

**Keywords:** Extrapelvic endometriosis, Magnetic Resonance Imaging

## Abstract

*Teaching point:* Extrapelvic endometriosis involving pudendal and sciatic nerve may be a cause of lower limb pain.

## Case History

**Figure 1:**
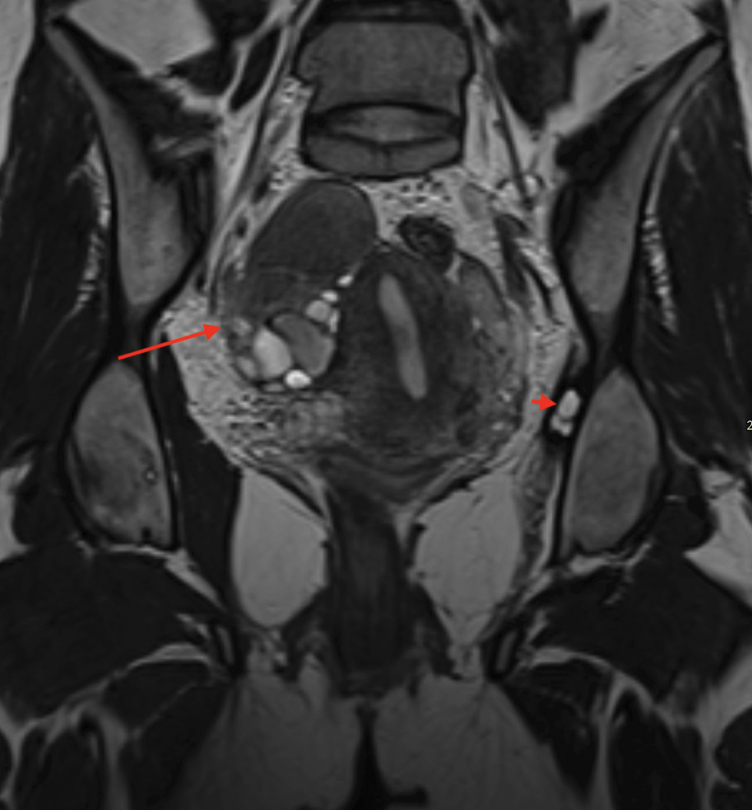
Coronal T2WI, showing a right adnexal endometrioma (long arrow) and a focus of extrapelvic endometriosis within the left postero-superior portion of obturator internus muscle (shot arrow).

**Figure 2:**
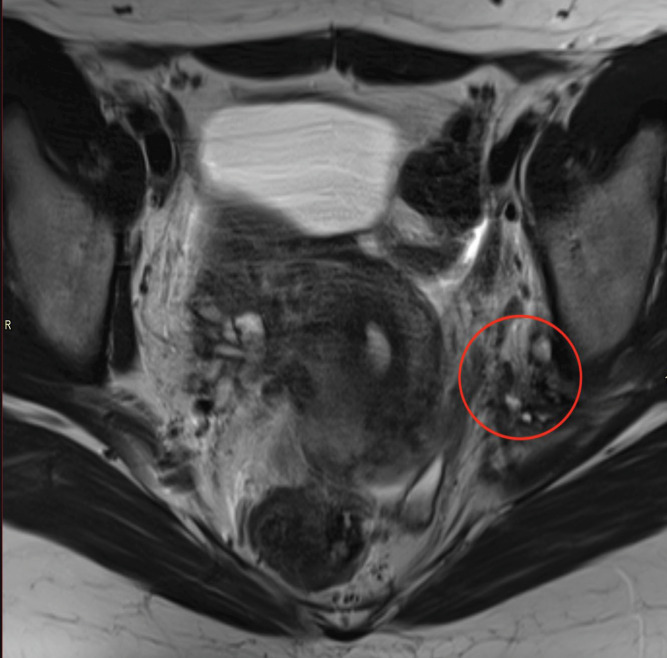
Axial T2WI, showing a 20 mm focus of endometriosis (encircled area) encasing and effacing both the sciatic and pudendal nerves.

**Figure 3:**
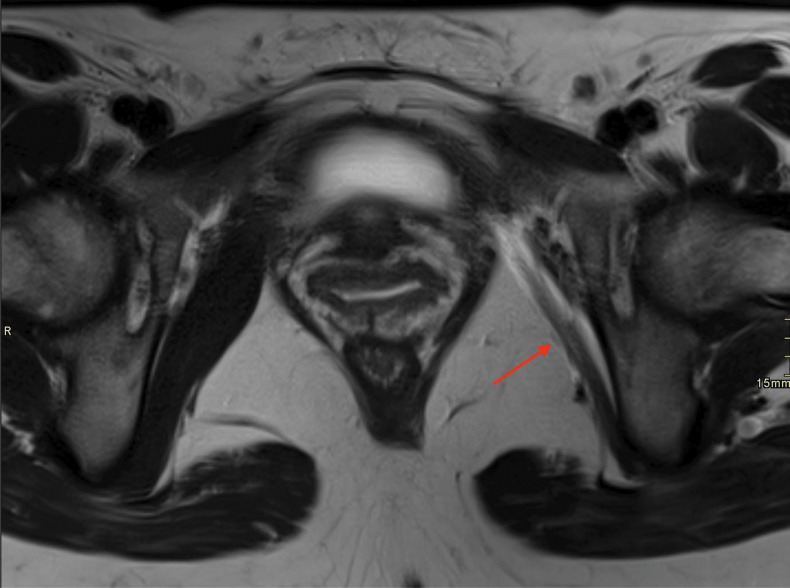
Axial T2WI, showing atrophy of the left obturator muscle.

A 29-year-old nulliparous, Caucasian woman with a 4-year history of infertility, presented to the emergency department complaining of an acute, strong abdominal pain. The physical examination revealed soreness in the lower right abdomen and suprapubic region. The biochemical findings were nonremarkable. Thorough medical history revealed that she suffered from severe dysmenorrhea and left lower limb pain during the last months. A pelvic ultrasound (US) revealed an adnexal cyst on the right ovary, with characteristics suggestive of an endometrioma, and a diagnostic laparoscopy was proposed with the possibility of performing drainage and biopsies. In the meantime, a pelvic magnetic resonance imaging (MRI) confirmed the presence of a multiloculated cyst on the right ovary of 65 mm in its largest dimension, hyperintense on T1WI, and demonstrating the “T2-shading sign” on T2WI (Figure 1, long arrow), suggestive of endometriosis. Furthermore, multiple foci of extrapelvic endometriosis were observed: a lesion of 10 mm within the left posterosuperior portion of obturator internus muscle (Figure 1, short arrow) and a larger lesion of 20 mm localized nearby the left greater sciatic foramen, causing fibrosis and retraction of the adjacent tissue and effacing and encasing the pudendal and sciatic nerve (Figure 2). Advanced atrophy of the left obturator muscle was observed, most likely a consequence of the involvement of both the muscle and of the nerve (Figure 3).

## Comments

Endometriosis [1] is defined by the presence of endometrium-like epithelium and/or stroma outside the uterine cavity. It represents one of the most challenging gynecological diseases, predominantly affecting the pelvis in women in their reproductive age, but should be considered as a systemic disease as it may occur in any organ. The main symptoms are chronic pelvic pain, dysmenorrhea, dyspareunia, and infertility. With extrapelvic endometriosis, symptoms may vary widely depending on the location, accounting for diagnostic confusion and, in many cases, diagnostic delay. Clinical examination, including vaginal examination when appropriate, may identify deep nodules or endometriomas; although, the diagnostic accuracy is low. Transvaginal US is the most common imaging modality used to evaluate suspected ovarian endometriomas. MRI is largely contributive to the evaluation of adnexal masses, deep infiltrating foci, and extrapelvic endometriosis. On MRI, endometriomas usually present as hyperintense masses on T1WI and, on T2WI, with a typically decreasing signal intensity from anterior to posterior, known as the “T2-shading sign.” Extrapelvic endometriosis presents as stellate fibrotic T2-hypointense strands with internal spots of active endometriosis. Extrapelvic endometriosis may infiltrate nerves, thus causing muscular denervation and subsequently muscular atrophy as in our case.

The therapeutical approach of (extrapelvic) endometriosis can either be medical and/or surgical. As for our patient, initial endometrioma drainage and biopsy through laparoscopy was chosen, followed by hormonal treatment leading to resolution of the symptoms.
